# Transcriptome analysis of a long-lived natural *Drosophila* variant: a prominent role of stress- and reproduction-genes in lifespan extension

**DOI:** 10.1186/1471-2164-13-167

**Published:** 2012-05-04

**Authors:** Agnieszka Doroszuk, Martijs J Jonker, Nicolien Pul, Timo M Breit, Bas J Zwaan

**Affiliations:** 1 Evolutionary Biology, Institute of Biology, Leiden University, Leiden, 2333 BE, The Netherlands; 2 MicroArray Department & Integrative Bioinformatics Unit, Swammerdam Institute for Life Sciences, University of Amsterdam, Amsterdam, 1098 XH, The Netherlands; 3 Netherlands Bioinformatics Centre, Nijmegen, The Netherlands; 4 Present address: Laboratory of Genetics, Plant Sciences, Wageningen University, Wageningen, 6708 PB, The Netherlands

**Keywords:** Ageing, Gene expression, Microarray, *Drosophila melanogaster*, Natural variation, Diet

## Abstract

**Background:**

While studying long-lived mutants has advanced our understanding of the processes involved in ageing, the mechanisms underlying natural variation in lifespan and ageing rate remain largely unknown. Here, we characterise genome-wide expression patterns of a long-lived, natural variant of *Drosophila melanogaster* resulting from selection for starvation resistance (SR) and compare it with normal-lived control flies (C). We do this at two time points representing middle age (90% survival) and old age (10% survival) respectively, in three adult diets (malnutrition, optimal food, and overfeeding).

**Results:**

We found profound differences between *Drosophila* lines in their age-related expression. Most of the age-associated changes in normal-lived flies were abrogated in long-lived *Drosophila*. The stress-related genes, including those involved in proteolysis and cytochrome P450, were generally higher expressed in SR flies and showed a smaller increase in expression with age compared to C flies. The genes involved in reproduction showed a lower expression in middle-aged SR than in C flies and, unlike C flies, a lack of their downregulation with age. Further, we found that malnutrition strongly affected age-associated transcript patterns overriding the differences between the lines. However, under less stressful dietary conditions, line and diet affected age-dependent expression similarly. Finally, we present lists of candidate markers of ageing and lifespan extension.

**Conclusions:**

Our study unveils transcriptional changes associated with lifespan extension in SR *Drosophila*. The results suggest that natural genetic variation for SR and lifespan can operate through similar transcriptional mechanisms as those of dietary restriction and life-extending mutations.

## Background

Ageing is a complex process controlled by genetic and environmental factors. Interventions that slow down or accelerate normal ageing, such as mutations in single genes are a powerful approach to investigate the mechanisms of ageing [1]. Studies using long-lived mutants identified genetic pathways and metabolic processes involved in lifespan determination. For example, the insulin/insulin-like growth factor-1 signalling (IIS) pathway was initially found in invertebrate models and was later demonstrated to be conserved among distant taxonomic groups including mammals [2]. While the studies on mutants have successfully unravelled many mechanistic details, the natural differences between individuals in lifespan and ageing rate in populations are poorly understood.

Studying natural genetic variants with different ageing phenotypes complements the findings obtained with mutants and validates their importance for natural populations [3]. Moreover, taking this approach can lead to identification of novel mechanisms, not revealed by mutant analysis [4]. One of the most straightforward and effective strategies focusing on natural genetic variation is the comparison of long-lived natural lines with those of regular lifespan. Long-lived lines can be obtained in multi-generation selection experiments, where direct selection for longevity or a trait associated with longevity leads to a divergence among the control and selected lines [5-7]. Because the natural phenotypic and genetic differences become magnified, the lines form a unique resource to help unravel the mechanisms behind natural variation in selected and associated traits. In particular, selected lines of *Drosophila melanogaster* have a long-standing record in studying trajectories of evolutionary responses of life-history traits as well as phenotypic, genetic and physiological relations among those traits [8]. For example, several selection experiments in this species demonstrated that if extended lifespan evolves, it is usually compromised by reduced fecundity or depression of other fitness components early in life (e.g. [9,10], but see [11,12]).

Over the last decade, genome-wide expression analysis tools such as microarray technology have been frequently applied to uncover the mechanisms underlying ageing. Transcriptomic studies have been conducted in many species, including invertebrate models [13-15], mouse and humans [16,17], either by comparing individuals across lifespan in longitudinal studies [1,14], or old and young tissues [17,18]. The derived age-related changes of transcript profiles, called “signatures of ageing”, were compared across species and a shared transcriptional profile of ageing was identified [19,20]. Transcriptomic analyses of long-lived *C. elegans daf-2* and *daf-16* mutants has confirmed the importance of insulin/IGF-1 pathway in regulating the genes involved in protection from stress for the control of lifespan and suggested a role for genes involved in mitochondrial function and fat metabolism [21,22]. It is uncertain whether these mechanisms underlie life extension in long-lived natural variants.

Environmental effects, such as diet, are often acknowledged to exert effects on ageing, but are seldom tested. A considerable interest has been dedicated towards investigating the effects of dietary and caloric restriction (DR and CR, respectively), which are known to extend lifespan in many species [23-27]. While these studies led to the identification of candidate mechanisms involved in a long-lived DR phenotype, they concern a very specific nutritional situation and do not necessarily reflect the magnitude and the character of other relevant dietary effects. A broader selection of dietary treatments in experimental analyses can help establishing the relative importance of nutrition versus genetic determinants of lifespan and ageing rate. Including several nutritional environments is especially valuable in analyses of lines that genetically differ in ageing-related traits as a result of evolutionary response to dietary conditions. Such an approach, by focusing on transcriptional reaction norms, allows discovering the processes and genes involved in adaptive changes involved in lifespan regulation.

**Figure 1:**
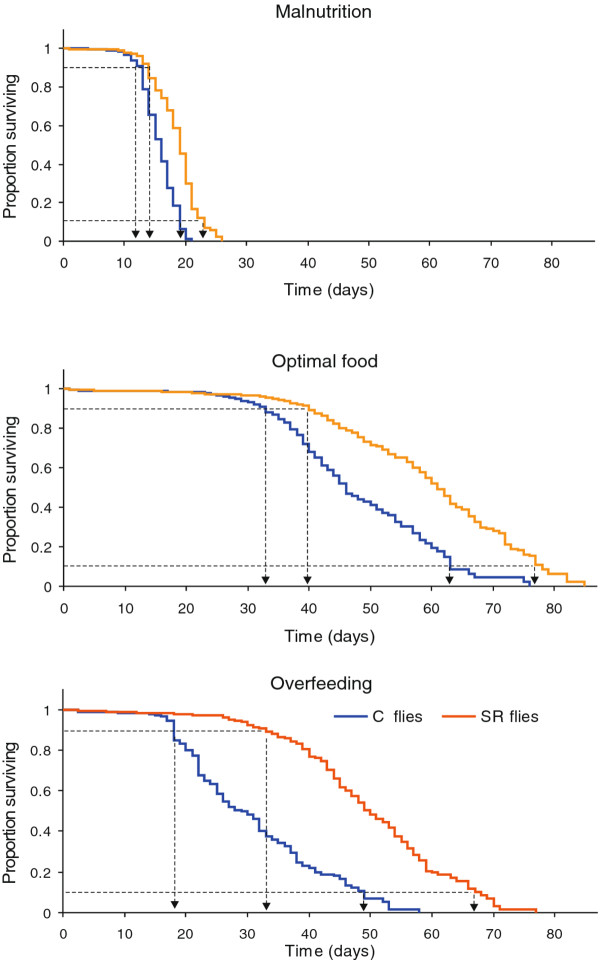
**Survival of C and SR flies under different diets.** Middle-aged and old flies were sampled after 10% and 90% of the cohort died, respectively. Arrows indicate the sampling points based on these demographic criteria.

Here, we present a transcriptomic study comparing age-related gene expression between females of long- and normal-lived *Drosophila melanogaster* natural variants. In our laboratory, we previously selected *Drosophila* for increased starvation resistance (SR) and maintained corresponding control lines under optimal food (C) [28]. The selection resulted in SR lines with not only elevated resistance to starvation but also extended lifespan under three other food levels as compared with the C flies. In this study, we focus on the SR line that showed a significant and proportional increase in both median and maximum lifespan (Figure 1), indicating a substantial change in the course of the ageing process. We investigate the effects of dietary interventions on ageing in both lines by including malnutrition and overfeeding next to the optimal food treatment in our transcriptomic analysis. This study provides, therefore, the first description of nutritional effects on transcriptional ageing phenotypes in differently ageing lines. Moreover, using this approach we were able to identify genes and processes most likely involved in lifespan extension in the SR flies.

## Results

To assess genome-wide expression levels, we cultured SR and C virgin female flies on three diets (malnutrition, optimal food and overfeeding) and sampled at the time points representing their middle and old age (after 10 and 90% of cohort died, respectively; Figure 1). In this way, we focused exclusively on the late ageing, in which the effects of ageing are directly linked with increased mortality rates. Our approach differs from the vast majority of *Drosophila* studies, which either focus on expression changes in relatively young adults or provide no explicit analysis of the changes in very old individuals. Addressing late ageing in model species is needed to provide a reference to human studies, which are often performed on individuals in the age classes characterized by high cumulative mortalities [29]. In particular, our time points were chosen to be comparable to the Leiden Longevity study [30], in which highly aged sib-pairs (10% survival) are compared to their children and their spouses (90% survival). We chose for demographic landmarks, rather than chronological age, to ensure that comparisons are made across corresponding age classes (middle-aged vs. old) of populations and treatments with different ageing rates. Moreover, demographic landmarks are more likely to represent physiological age, thus gene expression differences between physiological age classes can be treated as representative for health status and interpreted as molecular markers for the rate of ageing.

### Age-dependent gene expression in C flies under optimal diet

**Figure 2:**
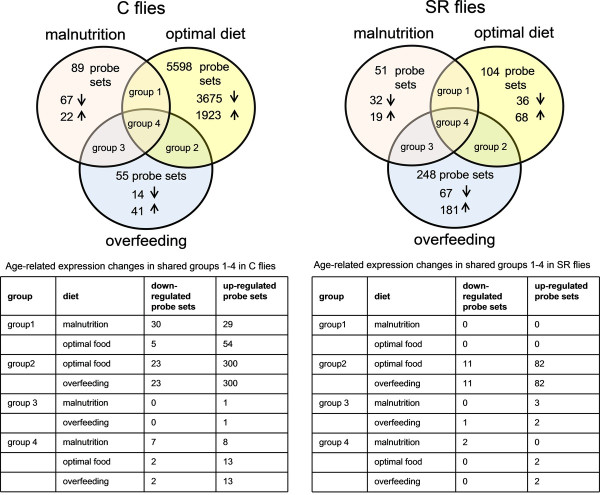
**Venn diagram representing probe sets with age-dependent expression.** Numbers of probe sets with age-dependent expression in C and SR flies under three different diets. Tables be low the diagrams display the numbers of probe sets overlapping in specific treatments and in the direction of change. Different treatment combinations are indicated as groups.

The R/Microarray Analysis of Variance MAANOVA indicated profound gene expression changes associated with age (Figure 2). Namely, 5995 probe sets representing 5378 genes (differentially expressed genes, DEGs) showed significantly different expression (38% of all genes) out of which 61% were downregulated. The effects of age were relatively high in comparison with other *Drosophila* studies. While males have been reported to have 4-19% of genes with age-dependent expression [15,31,32], females were shown to respond with 23% of genes [1]. The discrepancy between these and our results may arise partly from a higher accuracy of the expression values due to the applied experimental setup (4 replicates of 5 polled individuals) and less stringent significance level (FDR < 0.05), which we used to define DEGs, as well as from the differences in the age of the analyzed flies.

To increase statistical and explanatory power of the gene expression analysis, we used Gene Ontology (GO) annotation [33,34], which classifies genes into groups of similar molecular function, cellular localization or biological process, and performed gene enrichment analysis [35,36]. We found several pronounced features of the age-dependent expression in C flies. Firstly, genes associated with reproduction were significantly enriched in genes downregulated with age (Additional file 1). They included 148 genes involved in oogenesis and 25 genes involved in eggshell chorion assembly. This reduction in activity is clearly related to the decline of gonad function with age and seems to be a typical feature of transcript changes in ageing *Drosophila*[1,15]. Another group of genes repressed with age was associated with cell cycle, cell division and related categories such as DNA metabolism, DNA replication and chromatin organization. Products of the genes involved in these categories are predominantly active in the nucleus. Our study indicated also a profound reduction in transcript levels of genes involved in transcription, mRNA and tRNA metabolic processes, RNA splicing and ribosome biogenesis. Finally, we detected a substantial reduction in expression of genes involved in biosynthetic processes and intracellular transport of macromolecules. It is likely that the expression decline in all these categories is linked to the reduced reproduction activity in old flies, as *Drosophila* is primarily a post-mitotic organism and cell divisions in adults occur predominantly in relation to reproduction.

The genes involved in immune response and response to bacteria showed the most significant increases in transcript abundance at old age (Additional file 2). We detected the highest fold changes in genes encoding antimicrobial peptides including *DptB, AttA, AttC, AttD, Def* and several genes involved in peptidoglycan recognition (e.g. *PGRP-SB, PGRP-SD*). Strong induction of immune responses is among the major characteristics of ageing and was found in many distantly related species such as in *C. elegans* and mouse. Our results support this general pattern and demonstrate that the increase of activity of those genes is not specific to early adulthood but occurs also at later stages of ageing. Another category with increased transcript levels in old flies was proteolysis and related groups involved in peptidase and hydrolase activity. Earlier studies have indicated age-related expression changes of the genes related to protein metabolism, however with no consistent direction [15,32,37]. The age-dependent expression in C flies was also characterized in activation of genes involved in cytochrome P450, which suggests elevation of detoxification activities. Our analysis indicated expression increase in over 30 genes classified as belonging to this gene family. The detected age-related changes in expression of categories involved in metamorphosis including imaginal disc development and cellular morphogenesis are more difficult to interpret in the context of ageing.

The transcription changes of genes associated with reproduction, stress and immune response dominate age-dependent expression in our study, generally supporting earlier findings in *Drosophila* and other model species. Since the previous studies consider predominantly the alterations during early adulthood and our analyses focused on older age classes, we conclude that the transcription changes associated with those processes follow similar trends in early and late ageing.

### Age-dependent gene expression in SR flies under optimal diet

The long-lived SR flies showed much more similar transcript profiles across age classes as compared with the C flies (Figure 2). MAANOVA indicated that 199 probe sets representing 172 genes altered their expression with age (FDR < 0.05), thus the changes were one order of magnitude smaller than in C flies. Moreover, in contrast to the signature of ageing in C flies, where most of the genes reduced their expression with age, only 23% of DEGs were downregulated in old SR flies.

In the group of genes repressed in old SR flies, those related to visual perception were the most abundant (*P* < 0.0001; Additional file 3). In addition, the genes playing a role in muscle function including *Strn-Mlck* and *Fln* showed the largest reductions in transcript abundance with age. In contract to the pattern observed in C flies, the expression of genes involved in reproduction, cell cycle, DNA metabolism, transcription and ribosome biogenesis remained at a similar level across age classes. It suggests that the long-lived SR flies did not experience a typical decline of reproductive functions and biogenesis processes in the later stages of life. Direct comparison between SR and C flies showed that the maintenance of expression of those categories at a comparable level in ageing SR flies was coupled with their lower initial expression in middle-aged class as compared with those of C flies.

A greater similarity of C and SR flies in their age-dependent expression concerned the group of upregulated genes. Namely, ageing in SR flies was associated with activation of immune response (*P* < <0.001) and related processes such as peptidoglycan binding and peptidoglycan metabolic process. Similar to the signature of ageing of C flies, the genes with highest increases of transcript abundance encoded antimicrobial peptides (*AttA, AttC, DptB*). Also genes involved in proteolysis (*P* = 0.0087) with serine hydrolase and peptidase activity significantly increased their transcript abundance at old age (Additional file 4). Although these two major classes of genes showed elevated expression in old SR and C flies, their average expression levels differed between the lines. The pair-wise comparisons (SR vs. C flies) indicated that the stress-associated genes showed higher expression in middle-aged SR flies as compared with C flies of the same physiological age. In addition, ageing in SR flies was characterized by lower transcript abundances of those genes compared to C flies. It is likely that this pattern is related to a longer lifespan of SR flies, as the high expression of genes involved in stress responses has been previously shown to be associated with extended lifespan [22].

### Stress- and reproduction-related genes

**Figure 3:**
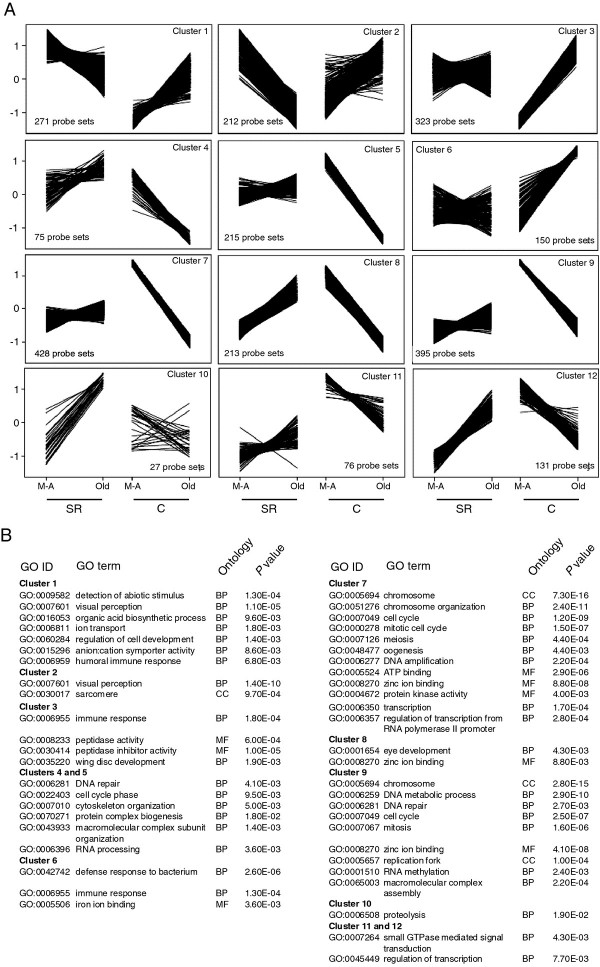
**Differential age-dependent expression between the C and SR*Drosophila.*** (**A**) Expression patterns of probe sets with differential age-related transcription between the *Drosophila* variants (defined by a significant age × line interaction). Probe sets are grouped into 12 co-expressed clusters using the K-means method. The expression values for each probe set were Z score transformed prior to the K-means analysis. (**B**) Significantly enriched GO categories in the identified probe set groups (BP: biological process, CC: cellular component, MF: molecular function).

To further investigate the differences between C and long-lived SR flies in their age-related expression, we focused on genes showing a line × age interaction for flies cultured on optimal diet. MAANOVA indicated 2511 probe sets representing 2270 genes with significant interactive effects (approximately 17% of all analyzed genes). We identified modules of co-expressed genes by applying K-means cluster analysis (Figure 3). From the 12 identified modules, two major groups could be distinguished: those with an increase of transcript abundance in old C flies (clusters 1, 2, 3 and 6) and those with repressed gene expression in these flies at the later age class (remaining clusters). The genes showing expression according to the first pattern were mostly involved in response to stimulus and stress responses. Especially, in clusters 3 and 6, where strong increases in gene activity in C flies were accompanied by only mild and inconsistent changes in SR flies, we found a high enrichment of immune response, proteolysis and cytochrome P450 genes. In the second major group of clusters (downregulated genes in old C flies), the dominant categories of genes included those related to DNA repair, DNA replication, translation with RNA, mRNA processing and RNA polymerase II transcription factor activity. Interestingly, despite the relatively large number of clusters within this major group, similar GO categories contributed to each cluster. This pattern indicates that while the expression of those genes is consistently and strongly repressed in old C flies, it can either increase or remain at a similar level in SR flies.

Our analyses strongly suggest that the difference in ageing phenotype between the *Drosophila* SR and C variants is associated with major changes in expression of genes involved in body maintenance, including biotic and abiotic stress responses, and genes related to cell cycle, transcription, protein synthesis, and reproduction. Because in adult fruit flies the last group of processes relates mainly to the reproduction-related functions, we will use the term “reproduction-related” genes to refer to this broader class of genes. The line × age interaction analysis suggest that these two major groups, stress- and reproduction-related genes, have an opposite age-related patterns of expression in both lines.

**Figure 4:**
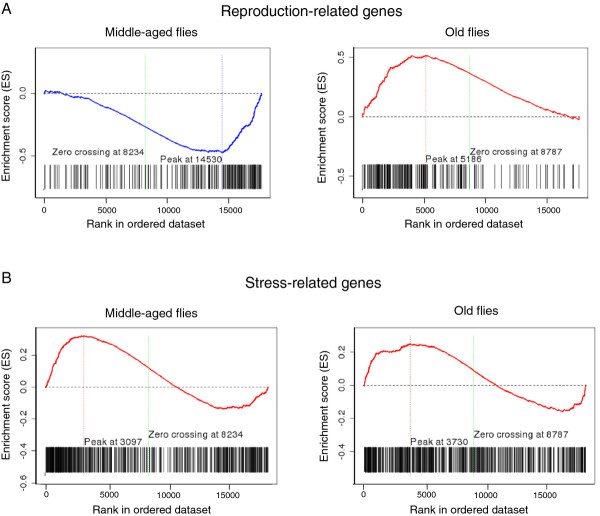
**Gene Set Enrichment Analysis with stress- and reproduction-related gene sets.** We applied GSEA to test whether the reproduction- and stress- gene sets are enriched in the rank-ordered list of genes for the comparison between C and SR flies. For each test, GSEA provides an enrichment score (ES) that measures the degree of enrichment of the gene set at the top or bottom of a rank-ordered gene list derived from the data set. A nominal *P* value was used to assess the significance of the ES. Vertical lines indicate the position of each of the genes comprising the stress and reproduction gene set. The figure panels show the plots of the running sum for the two gene sets: (**A**) reproduction and (**B**) stress gene set, respectively.

In order to test whether these major patterns of transcript changes are directly related to stress and reproduction functions, we performed a Gene Set Enrichment Analysis (GSEA) [34]. This analysis considers groups of genes linked together in functional pathways rather than single transcripts. We tested whether the sets of genes, whose activities are related either to reproduction or to stress, are enriched in SR relative to C flies (or vice versa) on optimal diet. We found that reproduction genes were significantly enriched in middle-aged C flies (*P* < < 0.001) and in old SR flies (*P* < < 0.001; Figure 4). The stress genes were enriched in SR flies in both age classes, however the enrichment shows a higher significance level in middle-aged flies (*P* < < 0.0001 and *P* = 0.00011, respectively). Also, it needs to be noted that while most of the stress genes showed higher expression in SR flies, some of genes in this gene set were higher expressed in C flies. These results indicate clearly an opposed regulation of reproduction-related genes across age classes in both *Drosophila* lines and an upregulation of stress-related genes in SR flies that weakens at older age. Overall, the GSEA results support the findings obtained with the K-means analysis, indicate that C and SR flies diverged profoundly in their expression of stress- and reproduction-related genes, and suggest the role of those gene classes in lifespan extension.

### Effects of diet

**Figure 5:**
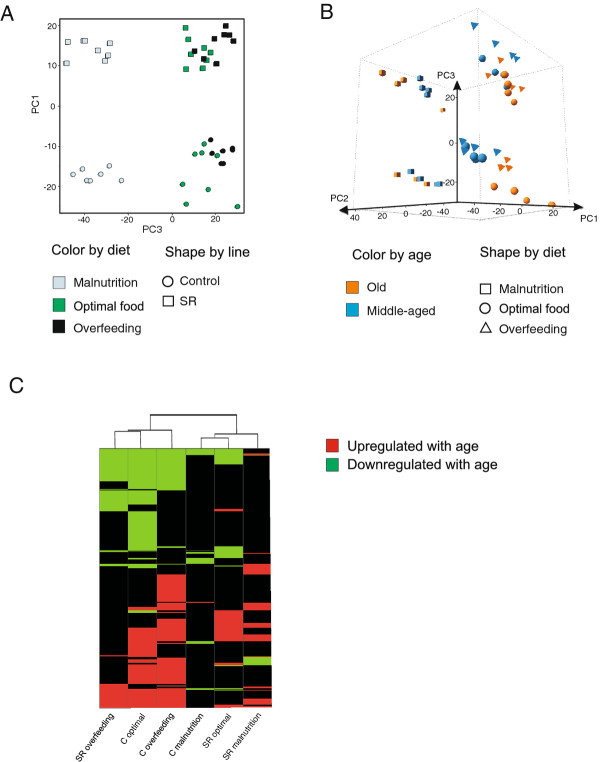
**Principal component analysis and the heat map of the expression data.** (**A**) Patterns of variation are presented in the subspace of the first and the third principal components (PC1 and PC3, respectively). (**B**) Patterns of variation are presented in the subspace of the first, second and third PCs. (**C**) Age-related expression at the level of GO categories for C and SR flies under all dietary conditions (gene categories up- or downregulated in the old flies).

Diet strongly affected gene expression in the analyzed *Drosophila* lines. Principal component (PC) analysis indicated that the first principal component (PC1) related to diet and explained 32% of the total variation in transcript abundances in our experiment (Figure 5A). The malnutrition samples were strongly divergent from those on optimal and overfeeding diets. *Drosophila* variant was associated with 11% of variation, and was represented by PC3. While for the second PC (21% of variation explained) no direct link with any single experimental factor was apparent, it is likely to be associated with the interactive effect of age, line and food treatments (Figure 5B). For example, while C flies showed a clear separation with age in optimal food and overfeeding treatment for PC2, the separation for SR flies was less apparent. These results were consistent with the results of MAANOVA, where the main experimental treatments were tested (averaged over the other experimental factors). The analysis indicated 7762 probe sets with expression levels changed in response to diet, 3261 probe sets differentially expressed in different *Drosophila* lines and 2510 probe sets with age related changes.

Diet also influenced age-related expression patterns. We found that the number of genes with age-dependent expression changes on malnutrition diet was one order of magnitude lower than those of on optimal food (Figure 2). Functional interpretation in both *Drosophila* variants revealed rather an inconsistent image of the age-associated processes under malnutrition. In C flies, the list of downregulated genes with age included genes involved in proteolysis and citrate cycle (*P* < < 0.001 and *P* = 0.009, respectively), while the categories involving drug metabolism and glutathione metabolism (*P* = 0.05 and *P* = 0.005, respectively) were enriched in the gene list with higher expression in old flies. Although the number of genes with age-dependent expression in SR flies was too low to deliver significant results of functional annotation clustering, we found that genes with reduced transcript levels in old flies included those involved in proteolysis, reproduction and lipid metabolism. Genes upregulated with age included those associated with transcription and RNA processing. In general, although our results indicate reduced effects of age for the flies on the malnutrition treatment, this pattern likely reflects stress of malnutrition dominating gene expression profiles in both age classes rather than an actual change in ageing processes. A similar effect is likely to be responsible for a high similarity of age-dependent expression in C and SR flies under malnutrition, as indicated by hierarchical clustering (Figure 5C). This interpretation is supported by the observations that malnutrition is associated with high mortality rates (Figure 1) and that gene categories related to stress response, including genes involved in detection of abiotic stimulus (*P <* < 0.0001), ion transport (*P* < < 0.0001) response to nutrient levels (*P* = 0.0009), were enriched in the group of genes upregulated under malnutrition condition at both age classes (Additional files 5 and 6).

Under overfeeding conditions, age-related expression changes in both *Drosophila* lines were similar to those of C flies on optimal diet (Figure 5C). We found a substantial overlap between these food treatments in the genes affected by age. More than 80% of probe sets detected in C flies under overfeeding treatment were also found to be differentially expressed under optimal diet. Moreover, the direction of the changes was in all the cases the same (Figure 2). Many gene categories identified in the signature of ageing of C flies, including proteolysis (*P* = 0.009), response to bacteria (*P* = 0.0005) and imaginal disc development (*P* < < 0.0001), were also detected in the overfeeding treatment. Interestingly, overfed C and SR flies showed more similar age-dependant expression than when under optimal diet (Figure 5C`). These results indicate that diet strongly modulates expression changes associated with age and that this modulation can be different for different genotypes. Moreover, they imply that similar age-dependent expression might arise either by the influence of genetic (different variant) or environmental (diet) determinants.

### Age-independent differences between C and SR flies

To address age-independent differences between the *Drosophila* variants, we focused on genes that show a consistent up- or downregulation in both middle-aged and old SR flies. Under the optimal diet, 240 probe sets representing 220 genes showed a higher expression in SR than in C flies. This list included genes involved in drug metabolism by cytochrome P450 (*P* = 0.001), glutathione metabolism (*P* = 0.004) and polysaccharide metabolic processes (*P* = 0.006). The first two categories relate to stress response, which suggests that an overall higher expression of these genes in SR flies is related to their increased starvation resistance. The enrichment analysis of the list of 135 downregulated probe sets representing 127 genes did not return significant functional categories. We analysed whether the differences between the lines detected under the optimal diet are also present in the other food conditions. We extracted probe sets showing consistent differences under all dietary conditions (Figure S1 in Additional file 7). They included 55 and 41 probe sets up- and downregulated is SR flies, respectively. The upregulated group contained genes involved in glutathione metabolism (*P* = 0.008), drug metabolism by cytochrome P450 (*P* = 0.02) and in phagocytosis (*P* = 0.01) indicating that a part of stress responses was activated in SR flies independently of diet and age. As before, for downregulated genes in SR flies, no significant functional categories could be identified.

### Candidate markers of ageing and candidate genes for lifespan extension

Genes that show age-related changes in expression and are unaffected by diet can be considered candidate markers of ageing. To identify them, we extracted genes with age-associated expression patterns for C flies similar on optimal and overfeeding diets. Since, under malnutrition conditions, as noted above, transcription patterns were dominated by stress responses, we did not include these measurements. For *P* < 0.01, the analysis revealed 266 candidate marker genes (Additional file 8), out of which 255 were upregulated with age. The categories enriched in this group included response to bacteria (*P* < 0.0001) with representing genes *pirk, PGRP-SA, PGRP-SC1, Tsf3, TepIII, TepIV, AttC*, proteolysis (*P* < 0.009) and imaginal disk development (*P* = 0.0006). Markers of ageing are predicted to show age-related transcriptional responses regardless of genotype. Therefore, in our more conservative approach, we considered the subset of candidate markers that show similar responses across the diets and, at the same time, also across the *Drosophila* variants. The analysis revealed a small group of 21 genes fulfilling these criteria (Additional file 9). All the genes were upregulated with age, 11 were annotated and included four genes involved in immune response: *PGRP-SA, vir-1, AttC and pirk.*

Genes that show age-dependent expression in C flies, but not in SR flies are predicted to be the candidate markers of healthy or delayed ageing. We identified those genes using the additional criterion of consistent patterns across the optimal and overfeeding diets. There were 57 genes upregulated with age in C flies that showed no changes in SR flies (Additional file 10). Genes involved in proteolysis including six genes encoding serine-type peptidases and genes involved in immune response (*AttA, Def, DptB* and *dro2*) showed the most statistically significant enrichment in this gene list (*P* = 0.019 and *P* = 0.015, respectively). Also, we detected four genes involved in drug metabolism while eight genes encode proteins of unknown functions. Twenty five genes showed a lower expression in old C flies, but no changes in SR flies (Additional file 10). Candidates with this pattern of expression are involved in RNA processing and chromosome organization (CG11837, *C12.1*, CG3709 and *Cap-H2*) and proteolysis (two genes with metaloendopeptidase activity and one gene with serine-type peptidase). Finally, we detected two genes of which products are active in mitochondria including mitochondrial transcription factor (*TFAM*) and CG15434, gene involved in oxidative phosphorylation.

## Discussion

The main objective of our study was to identify the mechanisms underlying lifespan extension in SR *Drosophila*. We used age-dependent gene expression of C flies under optimal food as a reference for interpretation of genetic and environmental effects on transcription. Along with providing a suitable reference, the results on C flies contribute to the understanding of the processes associated with late stages of normal ageing. This is a valuable aspect of this study, as most of the previous *Drosophila* research focused on expression changes occurring in early adulthood (but see [38]).

We found that age-dependent expression in C flies involved many processes already known to be associated with ageing. Nearly all functional categories upregulated with age in these flies including response to bacteria, innate immunity, proteolysis and drug metabolism have been previously indicated in other *Drosophila* transcriptomic analyses [15,31,39]. Our finding that genes related to reproduction decrease transcript abundances with age corresponds with earlier observations in *Drosophila*[1,15]. Finally, a downregulation of genes associated with cell cycle, DNA and RNA metabolism and transcription in old C flies resembles declines in these gene categories in other model species [40,41]. A relatively high similarity of our findings on late ageing and those on early ageing published previously suggests that most of the age-associated processes exert their effects during the whole adult stage. The only significant exception from this pattern concerns the genes involved in energy metabolism including glycolysis, tricarboxylic acid cycle, oxidative phosphorylation and electron transport. While a repression of oxidative metabolism genes is considered one of the most prevalent molecular features of ageing [1,20,31,42], we found no indication of changes in this gene class. This discrepancy is related to the fact that a sharp decrease in expression of the energy metabolism genes occurs in young adults [20,39] and late ageing is not characterized by their further downregulation.

Long-lived SR flies strikingly differed from C flies in their age-dependent expression. The first major difference between the lines concerns the number of genes differentially expressed with age: a vast majority of the changes associated with age in C flies were abrogated in SR flies. Such large discrepancy could potentially relate to confounding factors associated with experimental procedures rather than biology. However, the experiment-wise analyses of variation patterns in gene expression and the frequency of present and absent calls supported a biological origin of the differences (Figure S2 in Additional file 11).

Not only the magnitude of age-dependent expression changes was much lower in SR flies, also the functional groups were affected differently by age in both lines indicating that selection has strongly influenced the transcriptional ageing phenotype. The comparison of the age-dependent expression between the lines together with the analysis of co-expressed genes showing a significant line × age interaction indicated that the processes related to reproduction and stress response showed the most divergent expression patterns between the lines. We chose for a broad interpretation of processes involved in reproduction- and stress-related responses. We consider the changes in GO terms related to cell cycle, mitosis, meiosis, replication, DNA and RNA metabolism, transcription and ribosome biogenesis as linked with reproductive activities. That is based on the observation that *Drosophila* is primarily a post-mitotic organism and cell divisions occur predominantly in relation to reproduction. Although *Drosophila* mid-gut is maintained by the proliferation of stem cells, our GSEA with reproduction-related gene set based on an independent manipulative experiment [43] supports the main relation of cell division with reproduction activities. In a similar manner, we include immune response and response to bacteria to general stress-related responses. This interpretation follows the observation that many components of immune pathways are activated by abiotic stresses [44] and that upregulation of immune responses occurs in ageing *Drosophila* under sterile conditions, thus with no exposure to pathogens [38]. Also in the case of stress-related genes, our reasoning is supported by the result of the GSEA, where a stress-related gene set was created based on an independent data set [44].

We showed that genes involved in stress responses were generally higher expressed in SR flies and were characterized by smaller increases in expression with age as compared with those in C flies. Elevation in expression of this class of genes has been reported in many organisms whose lifespan was extended as a result of a mutation or caloric restriction [1,39,45-47], and has been proposed as one of the general mechanisms underlying lifespan extension [45,48]. We demonstrated that expression of reproduction-related genes was lower in SR than in C flies in the middle-age class and that it either increased with age or remained at a similar level, while showing a sharp decrease in C flies. These results follow the expectations driven from the studies at the phenotypic level, where extended lifespan is often associated with reduced or delayed fecundity (e.g. [12,49]). Also, our observations are generally in line with other gene expression studies, where lower expression levels of reproduction or cell-cycle genes were found in relation to longer lifespan ([1,45,50,51], but see [38]). The accumulating evidence from the expression studies of long-lived mutants and dietary restricted animals suggests that these alterations can have a causative role in ageing. It is also supported by the findings that experimentally limited protein synthesis leads to an increased lifespan in *C. elegans* and *Saccharomyces cerevisiae*[52]. Overall, the lower influence of age on expression levels of these two gene classes observed in SR flies resembles the patterns recorded in DR organisms and long-lived lines [1,38]. This similarity suggests that abrogation of the age-dependent expression belongs to the common mechanisms underlying lifespan extension and that natural genetic variation in lifespan present in natural populations is likely to be associated with a different magnitude of age-related expression.

**Figure 6:**
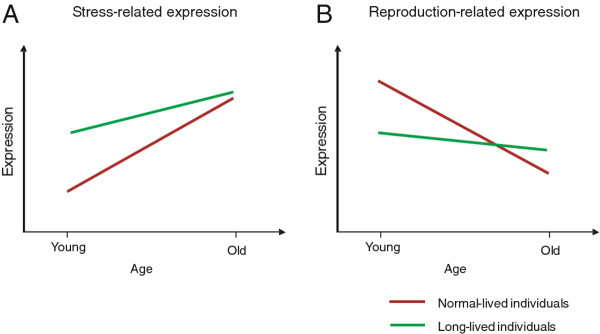
**Model of gene expression patterns associated with extended longevity.** Age-related gene expression of C and SR flies for (**A**) stress- and (**B**) reproduction-related genes. The expression pattern associated with normal ageing typically involves a strong upregulation of genes involved in stress responses. This reflects an activation of repair mechanisms counteracting the cellular damage associated with ageing. In long-lived individuals, genes involved in stress response and repair show generally a higher expression. The difference in expression is higher at younger age classes, as the normal-lived individuals upregulate those genes stronger with age. Another typical characteristic of age-dependent expression in normally-ageing individuals is a strong decline in genes involved in reproduction, which reflects ceasing reproductive activity at older age. Individuals with extended lifespan often show lower expression levels of those genes at younger age classes as compared to normal-lived controls, but their expression is maintained longer at relatively high levels. While the model generally reflects the existing expression data, some studies detected different patterns. For example, Sarup et al. [38] reported that the males of the lines selected for reproductive longevity showed on average higher expression of genes involved in cell cycle and biogenesis (reproduction-related genes) and a lower expression of the genes involved in immune functions (stress related genes). The proposed model might be more representative for females, where the reproductive activity is more directly reflected in gene expression. Also, the model is likely to refer to the expression patterns observed at the whole body level better than to those observed in specific tissues.

Figure 6 summarizes these observations and proposes a model of general age-dependent expression changes associated with life extension at the whole-body level. The model focuses on processes rather than specific pathways and genes. This follows the reasoning that differences in the ageing trajectory are associated with the changes at the process or function level conserved across taxa, and that similar effects on a process are likely to be achieved by the alterations in different pathways and/or genes [45]. The model visualizes how expected differences between the long-lived and normal-lived lines depend on the actual age of individuals. It also indicates that the interpretation of expression experiments will largely improve if multiple age classes are included and the determination of line × age interaction is possible. This type of data will ultimately contribute to establishing the generality of the patterns proposed in our model.

Next to the analysis of age-related expression in SR and C lines, we also present the results on age-independent differences between those lines. This analysis indicated that glutathione metabolism and cytochrome P450 were consistently upregulated in SR flies of both ages across all food levels. Glutathione and cytochrome P450 are involved in drug metabolism, detoxification and antioxidant responses, and their upregulation in flies with extended lifespan has been indicated as a result of DR [39] and other manipulations [48]. Sørensen et al. [51] also compared *Drosophila* selected for starvation resistance and for longevity with the corresponding controls. The analysis addressed age-independent differences among the selective regimes using multiple replicate lines per treatment. It showed that the selected lines (both regimes) downregulated genes involved in transcription, metabolism if nucleic molecules, spliceosome and glycolysis. These findings partially correspond to our results suggesting a general reduction of growth or cell division related processes in long-lived natural *Drosophila* variants. Recently, a reduced protein synthesis and an increased expression of glutathione in DR *Drosophila* have been linked by the trans-sulfuration pathway [53]. Higher lipid stores seem to be a common mechanism underlying increased SR [54] and changes in lipid metabolism have been indicated in genome-wide expression studies in long-lived mutants [55]. Our results have not indicated any major difference between the SR and C lines in this respect. These findings are in line with other studies on the natural variation in *Drosophila* lifespan, which did not indicate the changes in lipid metabolism at the level of gene expression [38,51,56].

Among the candidate markers of ageing identified in this study, genes involved in immune response showed the highest statistical significance. Genes involved in this gene category are likely candidates as they show strong age-related responses, which are consistent across *Drosophila* studies. For example, Pletcher et al. [39] also identified this class of genes as candidate markers of ageing and the comparison of our candidate markers with those obtained in the study by Sarup et al. [38] resulted in two overlapping genes: *pirk* and CG3036, out of which the first is involved in immune response. While the genes encoding imaginal disc growth factors have been identified as candidate markers of ageing [39], it is difficult to interpret their changes of expression in relation to ageing. They have been shown to interact with the insulin pathway to control growth and could possibly indicate the involvement of the c-Jun-N-terminal kinase (JNK) pathway [15].

Candidate markers of healthy ageing or extended longevity include genes belonging to the categories showing most divergent age-related expression between the *Drosophila* lines. This group of candidates was similar to those reported for DR flies [39], as both studies indicated genes encoding serine proteases and immune related genes. There is growing evidence that the molecular pathways underlying immune responses and lifespan are interlinked and it has been hypothesized that the immune system plays a major role in ageing and lifespan determination [57]. For example, long-lived *C. elegans* and *Drosophila* mutants were shown to have an improved tolerance to bacterial pathogens [58,59]. On the other hand, DR was not found to influence pathogen resistance, despite showing a significant effect on many immunity-related genes [59]. The strong effects on genes involved in immunity detected in this study, generally support an important role of immune system in lifespan determination. Finally, in the group of candidate markers of healthy ageing, we also detected two genes likely to be involved in energy metabolism. This is an interesting finding as the analyses performed separately for both lines and three diets did not indicate mitochondrial genes as an important group in age-associated gene expression changes.

While the main goal of this study was to identify the mechanisms underlying lifespan extension in SR *Drosophila,* it needs to be noted that the effects of selection for starvation resistance not always relate exclusively to longevity. Previous work indicated that this type of selection usually leads not only to SR and extended lifespan, but also to a lower fecundity, longer developmental time and a larger adult body mass [60]. Some studies suggest also that some processes affect SR and longevity independently or even antagonistically [61,62]. In general, lifespan extension whether acquired through a laboratory selection or a mutation, is likely to involve pleiotropic effects. Ultimately, understanding of life-history traits will require disentangling mechanistic relationships between those effects in the future studies.

In our experimental setup, the flies on the optimal food had the longest lifespan. Since in other studies, flies under DR display a maximum lifespan compared to other food levels, one may wonder whether the optimal food used in our study is not equivalent of DR treatment. Normally, DR involves restricting food by 30-40% in respect to the standard culturing conditions. In case of our experiment, optimal food is twice as concentrated as the standard medium used for culturing of C lines and the fly stock before the selection experiment. Thus, the optimal food treatment in this microarray experiment and DR treatment cannot be treated as each others’ equivalents. It needs to be noted that both C and SR lines showed a longer lifespan on the 2x medium (optimal food) than on the 0.5x, 1x, 2x, and 5x media (results on the1x medium are not shown). This suggests that, while selection resulted in a shift of the reaction norm elevation, the optimal diet remained similar in both lines.

We identified substantial effects of diet on age-related expression. Transcript patterns across different food levels could be considered as phenotypic reaction norms across environmental gradients. Following evolutionary interpretation of the changes in SR flies, the optimal performance of starvation adapted SR flies is expected to be shifted towards lower food levels and their age-related gene expression under malnutrition is predicted to resemble those of C flies under optimal food. At the same time, SR flies under control food should resemble overfed C flies. Our results did not follow these patterns. Therefore, adaptation to starvation and extended lifespan in SR flies are not associated with a shift of transcriptional reaction norm according to these evolutionary predictions. The clustering results of the heat map (Figure 5C) were more influenced by the *number* of age-related changes rather than their *character*. Consequently, the age-related patterns for C and SR flies under malnutrition characterized by a low number of changes due to the overruling stress-related expression appeared similar to ageing of SR flies under optimal food, where the age-associated changes were abrogated as a result of selection. In general, our analysis showed that diet effects were stronger compared to genetic effects (*Drosophila* variant). This large impact on age-associated transcript patterns was, however, mainly a consequence of a very divergent malnutrition treatment. Under higher food levels, the genetic and food effects were of a comparable magnitude (Figure 5C).

## Conclusions

Genome-wide analysis of long-lived *Drosophila* has provided insight into transcriptional changes associated with lifespan extension due to a natural genetic component. Our results indicated substantial changes in age-dependent expression of genes involved in reproduction and stress responses in SR flies. The direction and character of the changes resembled those observed in DR organisms and long-lived mutants suggesting common general mechanisms of lifespan determination at the process level. The analysis of diet-independent differences between the long- and normal-lived *Drosophila* allowed us to identify candidate markers of ageing and healthy ageing (extended lifespan). Additionally, our extensive experimental set-up enabled us to assess the contributions of environmental and genetic factors to age-dependent expression and establish the links between ageing and diet in determining transcriptional patterns. Overall, our results contribute to the understanding of the mechanisms underlying natural variation in lifespan and ageing rate.

## Methods

### Drosophila lines and microarray experiment

Previously, our laboratory has selected four lines for increased starvation resistance (SR) and maintained two control lines (C) in a 20 generation experiment [28]. We chose a single replicate line of each selection regime to perform the microarray experiment. The chosen SR line showed not only elevated resistance to starvation but also a consistent increase in lifespan across three diets in comparison with the C lines (Figure 1). Both, the median and maximum lifespan increased, strongly suggesting a delay in the ageing process. Since the lines diverged as a result of selection acting predominantly on standing genetic variation of the ancestor population, they both constitute natural *Drosophila* variants.

For the microarray experiment, the larvae were raised in vials (100 individuals per vial) with standard medium at 25°C and 60% humidity and sexed within eight hours post-eclosion. Adult virgin females were placed in vials (five individuals per vial) with media where yeast and sugar content was manipulated to obtain diet treatments representing malnutrition, optimal food and overfeeding (in total, 600 females per line and food level). We used three levels of adult nutrition that were predicted to induce different life-history phenotypes, but not to cause extreme stress [28] (optimal food – maximum lifespan, malnutrition and overfeeding exerted negative lifespan effects; Figure 1). The concentrations of yeast and sugar were relative to those of the standard medium (20 g agar, 9 g kalmus, 10 ml nipagin, 50 g saccharose and 35 g of granulated yeast per litre water). We used 0.5x concentration for the malnutrition, 2x for optimal food and 5x for the overfeeding treatments. The malnutrition treatment caused substantially less severe stress than the starvation treatment used during the selection experiment.

The flies were transferred to the vials with fresh medium every week. The survival was monitored for all line-diet combinations. We collected the middle-aged flies and old flies after 10% and 90% of the cohort died, respectively. For each treatment, four replicate samples were collected, each composed of five whole females snap-frozen in liquid nitrogen. This approach was chosen to minimise the possible bias due to the stochastic effects of late ageing. We extracted whole-body RNA from 48 samples (2 lines × 3 diets × 2 age classes × 4 replicates) using the NucleoSpin RNA II kit (Macherey & Nagel). The following steps including sample amplification, biotin labelling using the Ambion standard protocols, hybridization on Affymetrix Drosophila 2.0 GeneChip and readouts were performed by ServiceXS [http://www.servicexs.com].

### Data processing and analysis

We performed a set of quality control checks, i.e. visual inspection of the scans, RNA degradation analysis, examining the consistency among the replicated samples by principal component (PC) analysis, testing against criteria for signal to noise ratios and MA plot inspection. Two arrays of unsatisfactory quality were excluded from the further analysis. The probe level data of the remaining 46 arrays was summarized using the robust multi-array average (RMA) algorithm [63]. The data quality check and the subsequent analyses were performed using R (version 2.7.0) and Bioconductor [64].

Significant differences in expression were determined using ANOVA with MAANOVA package [65]. The permutation-based Empirical Beyes test (2000 random permutations) was used for hypothesis testing . To account for multiple testing, *p* values were adjusted to represent a false discovery rate (FDR) of 5% [66]. Seven contrast analyses were performed to detect differential gene expression. (i) test to determine general effects of experimental treatments including diet, age and line, (ii) test to identify the differences between the old and middle-aged C flies on optimal diet representing their age-dependent expression, (iii) test to detect the differences between old and middle-aged SR flies on optimal diet representing their age-dependent expression, (iv) test to find differences between middle-aged C and SR flies on optimal diet, (v) test to determine differences between old C and SR flies on optimal diet, (vi) test to identify differences in age-related expression between C and SR flies on optimal diet (age × line interaction tested), (vii) test to determine age-independent differences between old C and SR flies on three diets. Each analysis thus implemented a different contrast or a set of contrasts to focus on the influence of specific factors (or factor combinations) on transcript abundance.

Clustering of the expression patterns of the genes showing a significant age × line interaction (*P* < 0.05, FDR < 0.05) was performed using K-means algorithm (K = 12, Euclidian distance). In order to normalize the expression levels across the samples, the probe set intensities were Z score transformed prior to the K-means analysis.

We combined the information on differentially expressed genes and the Gene Ontology (GO) to relate the changes to biological processes, molecular function and cellular component. The functional annotation and GO term enrichment were analyzed using Expression Analysis Systematic Explorer (EASE) implemented in DAVID 6.7 [35,36]. The biological interpretation of the results of contrast analyses was performed based on the following steps: Genes showing a significantly different expression (*P* < 0.05, FDR < 0.05) in the contrast analyses were subjected to gene enrichment analysis in DAVID. For each contrast, the genes were divided into up- and downregulated classes and submitted for functional annotation chart and clustering analyses. While the first of the analyses presents similar annotation categories repeatedly, the second one is grouping similar categories reducing the redundancy of the GO terms. The options for the functional annotation clustering were as follows: similarity term overlap: 4, initial group membership: 4, similarity threshold: 0.5, Final group membership: 4 and multiple linkage threshold: 0.5. For the age-dependent expression in C and SR flies, we report functional categories showing enrichment score > 3.0.

### Gene set enrichment analysis for reproduction- and stress- related gene sets

For the comparison between SR and C flies at middle- and old age classes, all genes were ranked according to their t statistic value from the contrast analyses. Reproduction-associated gene set was constructed based on two pair-wise comparisons presented in the study of Parisi et al. [43]: i) between the expression in ovaries versus expression in female body without gonads and ii) between the expression in ovaries versus the expression in ‘tud’ females (females without gonads as a result of a genetic manipulation). We derived a common set of upregulated genes (fold change > 2) as those whose activation is related to reproductive functions. The stress-related gene set was constructed using data from Girardot et al. [44] who exposed *Drosophila* to paraquat and H_2_O_2_, two oxidative stressors, and tunicamycin which induces endoplasmic reticulum stress. Genes showing a significant upregulation (*P* < 0.05) in any of these stress treatments in comparison to the control treatment were selected. Only the genes that showed consistent direction of the expression change across the stress treatments were included. After the step of the gene symbol conversion performed in **FlyBase**http://flybase.org/ and subsequent exclusion of the genes with no existing record in **FlyBase**, redrawn genes and genes with multiple gene symbols, we obtained the sets of 203 and 478 genes, for the reproduction- and stress-related genes respectively.

### Data deposition

The data presented in this publication have been deposited in NCBI's Gene Expression Omnibus and are accessible through GEO Series accession number GSE36582. http://www.ncbi.nlm.nih.gov/geo/query/acc.cgi?acc=GSE36582.

## Abbreviations

C, Control, normal-lived Drosophila; SR, Starvation resistant, long-lived Drosophila; IIS, Insulin/insulin-like growth factor-1 signalling pathway; DR, Dietary restriction; CR, Caloric restriction; MAANOVA, R/Microarray analysis of variance; DEG, Differentially expressed genes; GO, Gene ontology; GSEA, Gene set enrichment analysis; PC, Principal component; JNK, c-Jun-n-terminal kinase; RMA, Robust multi-array average; FDR, False discovery rate; EASE, Expression analysis systematic explorer.

## Competing interests

The authors declare that they have no competing interests.

## Authors’ contributions

AD analyzed the results, carried out bioinformatic analyses and wrote the paper; MJJ carried out bioinformatic analyses and provided critical discussions; NP performed the microarray experiment and collected data; TMB provided critical discussions; BJZ initiated the study, designed the experiment and wrote the paper. All authors read and approved the final manuscript.

## Supplementary Material

Additional file 1 Table presenting GO terms associated with genes downregulated with age for C flies on optimal diet. Click here for file

Additional file 2 Table presenting GO terms associated with genes upregulated with age for C flies on optimal diet. Click here for file

Additional file 3 Table presenting GO terms associated with genes downregulated with age for SR flies on optimal diet. Click here for file

Additional file 4 Table presenting GO terms associated with genes upregulated with age for SR flies on optimal diet. Click here for file

Additional file 5 Table presenting GO terms associated with genes upregulated at malnutrition by middle-aged C flies. Click here for file

Additional file 6 Table presenting GO terms associated with genes upregulated at malnutrition by old C flies. Click here for file

Additional file 7 Figure showing age-independent differences between C and SR flies. Click here for file

Additional file 8 Table presenting candidate markers of ageing. Click here for file

Additional file 9 Table presenting candidate markers of ageing (conservative selection). Click here for file

Additional file 10 Table presenting candidate markers of healthy ageing (extended longevity). Click here for file

Additional file 11 Figure showing the patterns of variation in gene expression across all combined treatments (experimental groups). Click here for file
