# Supplementary material for: Transcriptome analysis of a long-lived natural Drosophila variant: a prominent role of stress- and reproduction-genes in lifespan extension
Source: BMC Genomics. 2012 May 4;13:167. doi: 10.1186/1471-2164-13-167 (PMC3427046; doi:10.1186/1471-2164-13-167)
Supplement: Additional file 7 — Figure showing age-independent differences between C and SR flies. [file 1471-2164-13-167-S7.pdf]

Figure S1

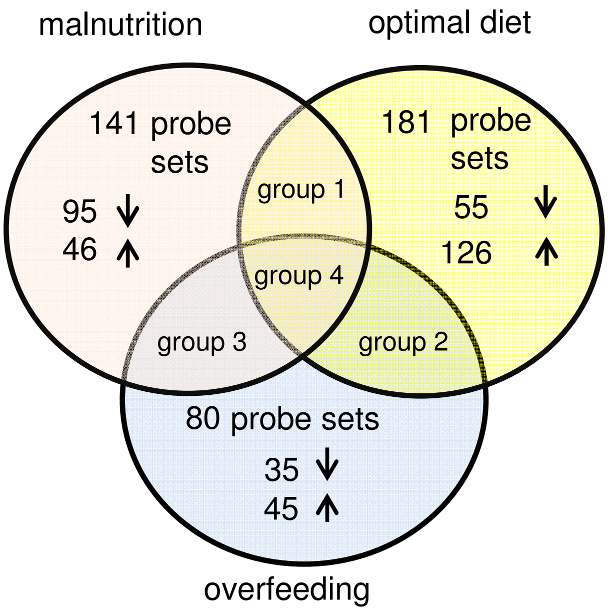

Expression differences between C and SR flies in shared groups 1-4

**group**	**down-regulated probe sets**	**up-regulated probe sets**
group1	18	29
group2	22	31
group 3	9	1
group 4	41	55

Figure S1. Age-independent differences between SR and C lines.
Venn diagram representing probe sets with differential expression between the *Drosophila* lines independent of age. Numbers of differentially expressed probe sets are presented for three different diets. The table next to the diagram displays the numbers of probe sets overlapping in specific treatments. Different treatment combinations are indicated as groups.
