# Supplementary material for: Transcriptome analysis of a long-lived natural Drosophila variant: a prominent role of stress- and reproduction-genes in lifespan extension
Source: BMC Genomics. 2012 May 4;13:167. doi: 10.1186/1471-2164-13-167 (PMC3427046; doi:10.1186/1471-2164-13-167)
Supplement: Additional file 11 — Figure showing the patterns of variation in gene expression across all combined treatments (experimental groups). [file 1471-2164-13-167-S11.pdf]

Figure S2

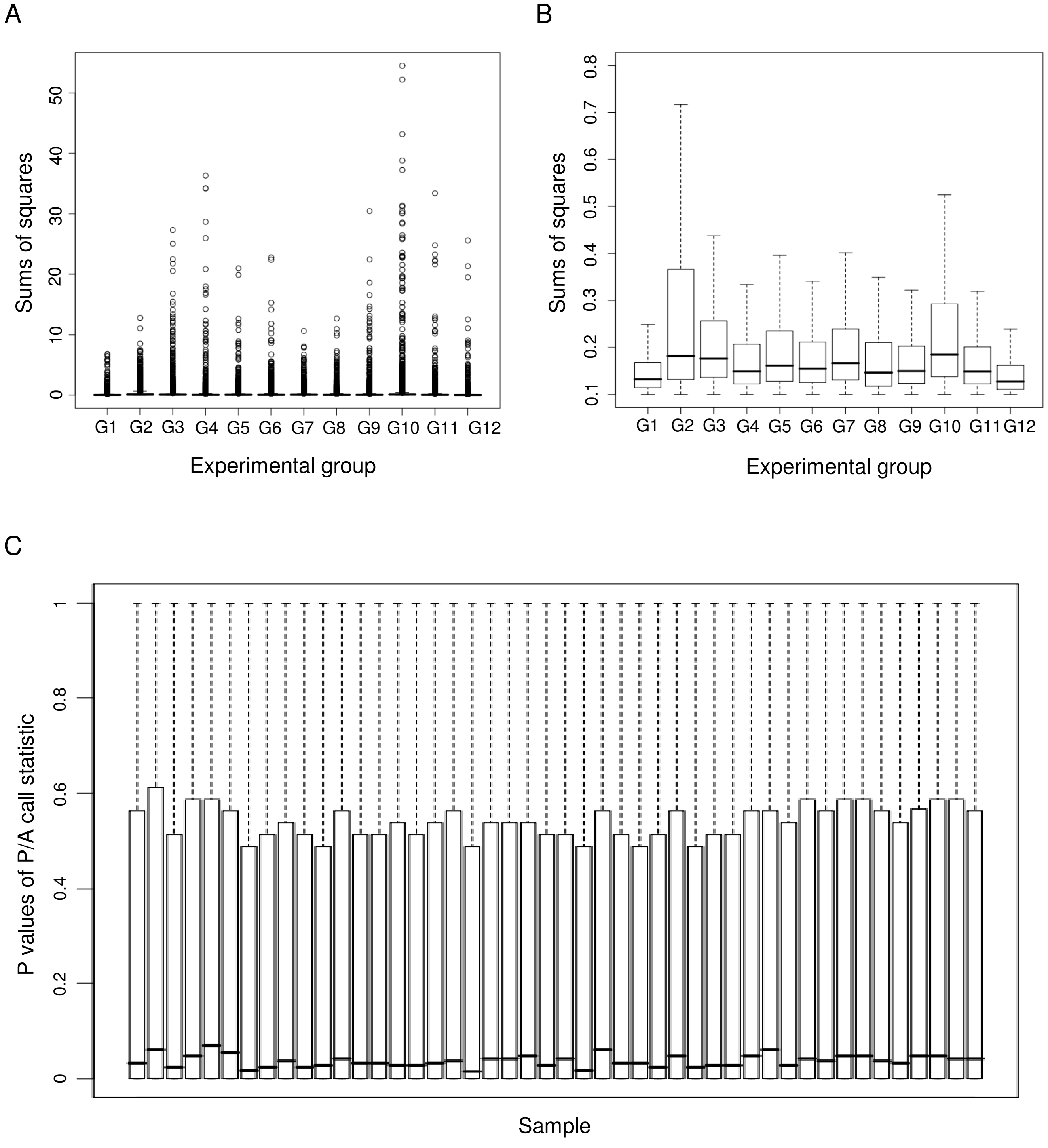

Figure S1 Patterns of variation in gene expression across all experimental groups.

Panel (A) shows the sums of squares with outliers and panel (B) depicts sums of squares without outliers for each experimental group. While the elevated levels of variation in expression could potentially arise as a result of environmental mismatch between SR phenotype and optimal diet and lead to a reduced

detection of DEGs between age classes, the comparison of sums of squares (measures of variation) across the experimental groups did not confirm this scenario. Panel (C) illustrates *P* values of the statistic to determine present/absent calls across all samples. Average higher *P* values and consequently lower frequencies of present calls in SR samples could potentially lead to reduced detection of DEGs across age classes. Our analysis excluded, however, this effect as an explanation for low age-related changes in gene expression of SR flies. Experimental groups: G1: Middle-aged SR flies on malnutrition diet; G2: Old SR flies on malnutrition diet; G3: Middle-aged SR flies on optimal diet; G4: Old SR flies on optimal diet; G5: Middle-aged SR flies on overfeeding diet; G6: Old SR flies on overfeeding diet; G7: Middle-aged C flies on malnutrition diet; G8: Old C flies on malnutrition diet; G9: Middle-aged C flies on optimal diet, G10: Old C flies on optimal diet, G11: Middle-aged C flies on overfeeding diet, G12: Old C flies on overfeeding diet,
